# Unpicking the Roles of DNA Damage Protein Kinases in Trypanosomatids

**DOI:** 10.3389/fcell.2021.636615

**Published:** 2021-08-06

**Authors:** Gabriel L. A. Silva, Luiz R. O. Tosi, Richard McCulloch, Jennifer Ann Black

**Affiliations:** ^1^The Wellcome Centre for Integrative Parasitology, Institute of Infection, Immunity and Inflammation, University of Glasgow, Glasgow, United Kingdom; ^2^Department of Cell and Molecular Biology, Ribeirão Preto Medical School, University of São Paulo, Ribeirão Preto, Brazil

**Keywords:** protein kinases, PIKK, DNA damage, DNA repair, kinetoplastids, trypanosomatids

## Abstract

To preserve genome integrity when faced with DNA lesions, cells activate and coordinate a multitude of DNA repair pathways to ensure timely error correction or tolerance, collectively called the DNA damage response (DDR). These interconnecting damage response pathways are molecular signal relays, with protein kinases (PKs) at the pinnacle. Focused efforts in model eukaryotes have revealed intricate aspects of DNA repair PK function, including how they direct DDR pathways and how repair reactions connect to wider cellular processes, including DNA replication and transcription. The Kinetoplastidae, including many parasites like *Trypanosoma* spp. and *Leishmania* spp. (causative agents of debilitating, neglected tropical infections), exhibit peculiarities in several core biological processes, including the predominance of multigenic transcription and the streamlining or repurposing of DNA repair pathways, such as the loss of non-homologous end joining and novel operation of nucleotide excision repair (NER). Very recent studies have implicated ATR and ATM kinases in the DDR of kinetoplastid parasites, whereas DNA-dependent protein kinase (DNA-PKcs) displays uncertain conservation, questioning what functions it fulfills. The wide range of genetic manipulation approaches in these organisms presents an opportunity to investigate DNA repair kinase roles in kinetoplastids and to ask if further kinases are involved. Furthermore, the availability of kinase inhibitory compounds, targeting numerous eukaryotic PKs, could allow us to test the suitability of DNA repair PKs as novel chemotherapeutic targets. Here, we will review recent advances in the study of trypanosomatid DNA repair kinases.

## Introduction

Numerous DNA lesions can form within a eukaryotic cell per day, each a potential threat to genome stability (Tubbs and Nussenzweig, 2017). Genome damage can arise from a myriad of sources, including exposure to mutagenic agents, such as radiation, and endogenous cellular processes such as DNA replication and metabolism. Lesions can form primarily on a single DNA strand, such as by the accumulation of unbase-paired single-stranded DNA (ssDNA), base adducts, oxidative damage, and mismatched bases, or can affect both stands, such as through double-stranded breaks (DSBs) and inter-strand cross-links. Ultimately, the persistence of all such damage can compromise high-fidelity genome transmission to future offspring, resulting in genetic diseases, decreased fitness, or lethality (O’Driscoll, 2012; Ribezzo et al., 2016; Chatterjee and Walker, 2017). Conserved across the Eukarya, a sophisticated network of pathways, collectively known as the DNA damage response (DDR), operate to safeguard the genome, acting hierarchically from lesion detection to resolution. At the heart of the DDR are evolutionarily conserved protein kinases (PKs) that act to orchestrate the repair of genome damage by signaling its presence and enacting the appropriate repair pathway *via* post-translational phosphorylation modifications to the hydroxyl groups of serine (S), threonine (T), or tyrosine (Y) residues on downstream factors. Additionally, DDR PKs also perform a range of non-catalytic functions, such as by the allosteric regulation of other kinases (Kung and Jura, 2016).

The DDR and its associated PK compliment are well-characterized in “model” eukaryotes, but in trypanosomatids, less is known. Trypanosomatids are parasitic members of the widespread and diverse Kinetoplastea class (Lukeš et al., 2018; Butenko et al., 2020) and cause neglected tropical diseases (NTDs) that disproportionally affect impoverished populations in the tropics and subtropics of the world. Human African Trypanosomiasis (*Trypanosoma brucei*), Leishmaniasis (*Leishmania* spp.), and Chagas disease (*Trypanosoma cruzi*) are three of 20 NTDs targeted by the World Health Organization (WHO) for eradication by 2030 (World Health Organization, 2020). These dixenous parasites transmit from arthropod vectors to mammalian hosts (for life cycles of each parasite, refer to Stuart et al., 2008), where they cause debilitating but distinct diseases of medical importance, which significantly impact the life quality of the infected individual and at-risk populations, and, combined, are responsible for ∼80,000 deaths each year (Torres-Guerrero et al., 2017; Büscher et al., 2017; Pérez-Molina and Molina, 2018).

Trypanosomatids are early branching eukaryotes, having emerged ∼500 million years ago, close to the time mammals emerged from other eukaryotes (Lukeš et al., 2014). As such, unusual aspects of the DDR, including during DNA repair, have been reported. For instance, classical non-homologous end-joining (c-NHEJ) activity required for DSB repair is lacking in these organisms (Burton et al., 2007; Nenarokova et al., 2019), with the result that DNA end-joining using regions of micro-homology (MMEJ) (Glover et al., 2011; Laffitte et al., 2016) or by single-strand annealing (SSA) (Glover and Horn, 2014; Zhang and Matlashewski, 2019) appears to assume greater prominence than in many organisms. In addition, nucleotide excision repair (NER) appears to have become functionally streamlined (Machado et al., 2014), most likely due to the ubiquity of multigenic transcription in kinetoplastids. Other peculiarities have recently emerged, with components of the 9-1-1 complex playing non-canonical roles in *Leishmania* genome replication, as facilitators of genomic plasticity (Damasceno et al., 2016, 2018). Moreover, DDR PK activity has been implicated in developmental transitions between host and vector (Baker et al., 2021) and as a driver of host immune evasion (Black et al., 2020). Thus, the trypanosomatid DDR and its associated PKs have potential for the discovery of novel biology and the prospect of parasite-specific drug targets.

Dysregulation of PK activity is commonly reported in human disease (Cell Signaling Technology, 2020), with over 80 small-molecule inhibitors approved for clinical use (Carles et al., 2018; MRC Protein Phosphorylation and Ubiquitylation Unit, U. of D, 2020; Roskoski, 2020). Thus, rather than developing novel compounds that target DDR PKs, an opportunity exists for the repurposing of small-molecule inhibitors as novel anti-parasitic treatments, particularly as the development of drugs targeting NTDs is routinely limited due to safety, efficacy, and funding, leaving many archaic and dangerous drugs at the forefront of treatments for the foreseeable future (Field et al., 2017; Bhattacharya et al., 2020). Additionally, using such small-molecule PK inhibitors could provide opportunities to investigate both the function and evolution of PKs, including those that act in the trypanosomatid DDR. Such an approach may be especially attractive for less genetically tractable trypanosomatids, like *T. cruzi* and *T. vivax*. Here, we will focus on trypanosomatid DNA damage-associated PKs and their reported functions, first discussing the known roles of canonical DDR PKs and then focusing on wider putative DDR PKs.

### PKs at the DDR Apex

**FIGURE 1:**
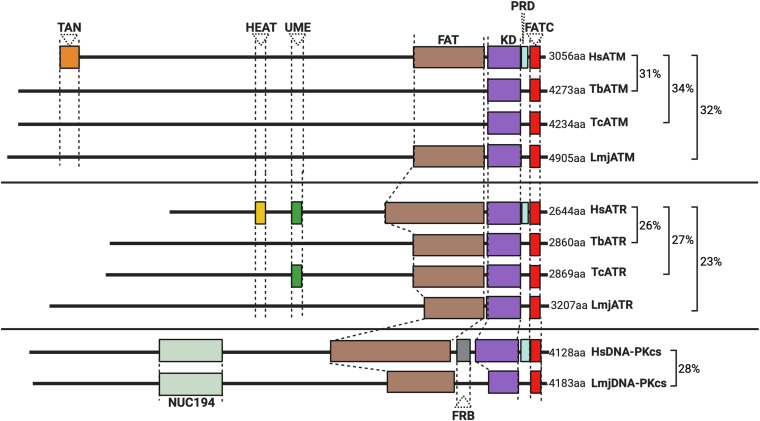
Schematic illustration of the predicted domain locations in Trypanosomatid PIKKs compared with their human homologs. Putative domains were identified using Pfam (http://pfam.xfam.org), Prosite (https://prosite.expasy.org), and Interpro (https://www.ebi.ac.uk/interpro/). Sequence similarity was determined using BLAST (Altschul et al., 1997), and all sequences from trypanosomatids are compared to the corresponding human kinase sequence. Gene IDs, the percentage identity, and the E value for each sequence are as follows: HsATM (AAB65827.1), TbATM (TbATM427_020008900; 31.47%, 2e-99), TcATM (TcCLB.509395.20; 33.95%, 7e-108), and LmjATM (LmjF.02.0120; 31.73%, 9e-94). HsATR (NP_001175.2), TbATR (Tb427_110165100; 26.14%, 7e-119), TcATR (TcBrA4_0103840; 27.17%, 2e-199), and LmjATR (LmjF.32.1460; 23.45%, 2e-97). HsDNA-PKcs (NP_008835.5), LmjDNA-PKcs (LmjF.36.2940; 27.58%, 4e-30).

PKs are specialized enzymes accounting for up to 3% of the encoded genes in a typical eukaryote (Hunter and Plowman, 1997; Manning et al., 2002; Zulawski et al., 2014). Two superfamilies of PKs exist including eukaryotic PKs (ePKs) and atypical PKs (aPKs), with nine subfamilies of ePKs described in most eukaryotes. The ePK structure is largely conserved among subfamilies, where an N-terminal lobe (composed primarily of β-sheets) is joined by a hinge-like region to a predominantly α-helical C-terminus, with the site of γ-phosphate transfer (the active site) located between these extremities (Hanks and Hunter, 1995) (for an extensive review on PK structure, refer to Taylor and Kornev, 2011). aPKs typically lack the catalytic region or domains characteristic of ePKs, yet among the aPKs, members of the phosphatidyl inositol 3′ kinase-related kinase (PIKK) family perform vital functions at the apex of the DDR. DNA-dependent Protein Kinase catalytic subunit (DNA-PKcs; absent from yeasts), ATR (Mec1 in budding yeast), and ATM (Tel1 in budding yeast) are large enzymes (up to 500 kDa in size) sharing structural similarities with lipid kinases within their C-terminal kinase domains (Figure 1). Flanking their kinase domains, they share several further conserved domains, including the FAT domain (FRAP, ATM, and TTRAP domain), a protein regulatory domain (PRD), a LST8-binding element (LBE) domain, and FAT-C domain downstream, all of which are required for kinase function and activity regulation (as reviewed by Imseng et al., 2018). The N-terminal regions of the PIKKs comprise much of their sequence and are arranged as “superhelices” (or α-solenoids), consisting of coiled Huntingtin, elongation factor 3, protein phosphatase 2A, and TOR1 (HEAT) repeats, which can modulate kinase activity (Mori et al., 2013; Luzwick et al., 2014; Chen et al., 2021). PIKKs typically phosphorylate substrates carrying an S/T motif followed downstream by a glutamate (Q) residue, with canonical activation of each PK occurring in a substrate-dependent manner. In addition, and as discussed below, each PK interacts with a number of non-kinase proteins to effect and modulate its activity.

**FIGURE 2:**
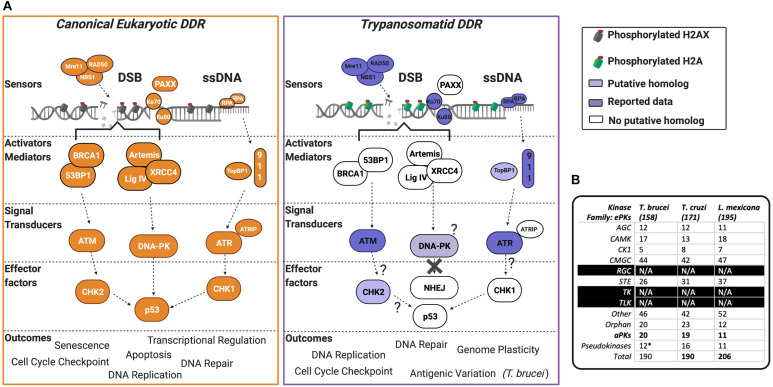
The PIKK-driven DDR pathways in Trypanosomatid parasites and the canonical Eukaryotic pathways. **(A)** A schematic illustration of a simplified eukaryotic DDR pathway (left) compared to known or predicted components of the trypanosomatid DDR pathway (right). Dark shaded factors indicate that functional characterization has been performed in one or more organisms. Light shading indicates limited data availability. White indicates no data are available or the factor is not present in the genome, as further illustrated by question marks. For more intricate details on eukaryotic DDR factors and pathways, we encourage the reader to refer to recent reviews (Alexander and Orr-Weaver, 2016; Blackford and Jackson, 2017; Wright et al., 2018; Sun et al., 2020; Zhao et al., 2020; Ghosh and Raghavan, 2021). DSB, double-stranded break; DDR, DNA damage response; ssDNA, single-stranded DNA. **(B)** Summary table of PKs and their associated families in *T. brucei*, *T. cruzi*, and *L. mexicana*. Data collated from Parsons et al. (2005), Jones et al. (2014), and Baker et al. (2021). (*) = the pseudokinases in *T. brucei* are included among the counts for the other families and their respective numbers have not been adjusted to remove pseudokinase family members. N/A = no kinases have been identified as members of these kinase families.

At least two of these three PIKKs are encoded in the genomes of *T. brucei*, *T. cruzi*, and *Leishmania.* In the following sections, we will describe ATM, DNA-PKcs, and ATR, and discuss their reported roles in trypanosomatids (Figure 2A shows a summary of the pathways these kinases act within).

### The ATM Kinase

In humans, low expression or inactivation of ATM causes ataxia-telangiectasia (A-T), a neurodegenerative syndrome associated with growth retardation, cancer predisposition, immune response deficiency, and genomic instability (Savitsky et al., 1995; Rothblum-Oviatt et al., 2016). Surprisingly, whereas murine ATM null mutants are viable (Barlow et al., 1996; Elson et al., 1996; Xu et al., 1996), kinase-dead mutants fail to survive past embryogenesis and show increased chromatid damage associated with replication stress (Daniel et al., 2012; Yamamoto et al., 2012, 2016). Thus, the inactive kinase likely inhibits other repair factors from carrying out their repair functions. ATM is activated by DSBs detected by the Mre11-Rad50-Nbs1 (MRN) complex (Figure 2A). MRN unwinds the helix and performs end-resection, exposing regions of ssDNA, which is pivotal for ATM recruitment and optimal activation (Lee and Paull, 2005). Full activation of ATM requires dissociation of the inactive dimeric form of the PK, with subsequent phosphorylation events triggering conformational changes that release one dimer and activate the other (Bakkenist and Kastan, 2003). Once active, ATM auto-phosphorylates and phosphorylates downstream substrates, including the variant histone H2AX (on serine-139) in higher eukaryotes to generate the genotoxic stress marker yH2AX (Burma et al., 2001). However, for many single-celled eukaryotes, for example, yeast (Downs et al., 2000), trypanosomatids (Glover and Horn, 2012), and the apicomplexan parasite *Plasmodium falciparum* (the etiological agent of malaria) (Manish et al., 2021), the equivalent ATM phosphorylation occurs on the core histone H2A. ATM can also activate p53 (a tumor suppressor protein) and other PKs, including the checkpoint kinase checkpoint 2 (CHK2), halting cell cycle progression at G_1_/S and G_2_/M and promoting DSB repair *via* NHEJ (an error-prone pathway) or homologous recombination (HR; a high fidelity pathway) (Awasthi et al., 2015). ATM also plays a role in telomere maintenance (Hande et al., 2001; Lee et al., 2015; Tong et al., 2015). ATM-deficient cells exhibit shortened telomeres linked to defective telomerase recruitment (an enzyme that extends telomeric sequences) (Ritchie et al., 1999; Lee and Paull, 2005; Tong et al., 2015). ATM also acts upon dysfunctional telomeres, which are a source of genomic instability, by eliciting a cell cycle checkpoint and cell senescence (D’Adda Di Fagagna et al., 2003).

The N-terminal region of the trypanosomatid ATM kinase is predicted to form an α-solenoid structure, accounting for ∼57% of the enzyme (Figure 1). A FATC regulatory domain and a C-terminal kinase domain typical of the PIKK family can also be detected. However, several domains are either absent or diverged in several trypanosomatids: a discernable FAT domain is absent in both *T. cruzi* and *T. brucei*, but present in *Leishmania*; TAN domains (required for telomeric maintenance and DSB repair activities in other eukaryotes; Seidel et al., 2008) and LBE domains also appear to be lacking in all trypanosomatid ATMs. When combined with the lack of identifiable phosphorylation sites in phosphoproteomic studies in *T. brucei* (Urbaniak et al., 2013), these domain variations suggest that the regulation of trypanosomatid ATM by phosphorylation is unclear and may even differ between related trypanosomatids.

RNA interference (RNAi)-mediated depletion of ATM in mammal-infective *T. brucei* initially revealed a lethal phenotype *in vitro* (Forsythe, 2012). However, more recent genetic screens (Jones et al., 2014; Stortz et al., 2017) suggest that *T. brucei* ATM may be non-essential in mammal-infective cells, though effects of ATM loss in tsetse stage *T. brucei* are unknown. Moreover, whereas in other eukaryotes ATM functions during DSB repair, this functionality has not been directly tested in *T. brucei*. Thus, how ATM operates in the context of the DDR across the *T. brucei* life cycle is unclear.

In *L. major*, ATM function has been investigated in promastigote (sandfly-infective) cells using the small molecule KU-55933 (da Silva R. B. et al., 2018), which inhibits ATM activity in human cells (Hickson et al., 2004). When promastigotes were exposed to a range of KU-55933 concentrations, a moderate slowing of parasite proliferation with little perturbation of the cell cycle progression was observed, even at high concentrations of the compound. Treatment with KU-55933 sensitized parasites to H_2_O_2_, implicating ATM kinase activity in tackling oxidative stress-derived lesions. Whether KU-55933 treatment induces a more generalized sensitivity to genotoxins requires further investigation, as we lack information about how selective this inhibitor is for ATM in trypanosomatids. In a recent study, an unexpected role for the ATM gene in *L. mexicana* was uncovered (Baker et al., 2021). Deletion of ATM in promastigotes prevented the establishment of infections in the sandfly vector, implicating ATM (and perhaps the wider DDR directed by the PK) in a previously unappreciated role in parasite transmission, though the basis for this defect is unexplained. In fact, in both these aspects of infectivity, the *L. major* ATM mutants are worthy of further study, given the inhibition data. *Leishmania* are intracellular parasites of mammals, developing within immune cells such as neutrophils and macrophages, which generate reactive oxygen species (ROS). One could speculate that loss of ATM may increase sensitivity to ROSs generated during development in the host cell, compromising parasite viability and thus transmission potential. If so, ATM may be a candidate target to block parasite transmission. To date, nothing has been reported about ATM function in *T. cruzi*.

As mentioned above, ATM phosphorylates histone H2A or H2AX in response to DNA damage. In trypanosomatids, damage-dependent phosphorylation occurs on the core histone H2A at residue Thr130 (Glover and Horn, 2012). Following genotoxin exposure, the yH2A signal can be detected either as a diffuse nuclear signal or as foci depending on the damaging agent, consistent with PK activity during the DDR. However, no work has shown that yH2A contributes to DNA damage repair, and it is unknown what PK is responsible for the phosphorylation, although, mutation of MRE11 abrogates the reaction (Dattani and Wilkinson, 2019). In addition, depletion of another DDR PK (ATR, discussed in a later section) increases yH2A levels (Black et al., 2020).

The principal downstream substrate of ATM is checkpoint kinase 2 (CHK2), which can induce a G_1_/S-phase cell cycle stall upon activation (Matsuoka et al., 2000). A CHK2-like protein has been identified in trypanosomatids, but no work has confirmed this PK as a bonafide CHK2 homolog (Genois et al., 2014). Another key substrate of ATM is p53, which is present in metazoans (Dos Santos et al., 2016) and some unicellular organisms (Lu et al., 2009; Bartas et al., 2020), though trypanosomatids appear to lack a p53 homolog. Thus, putative events downstream of trypanosomatid ATM are unknown. Loss of MRE11 or RAD50, the upstream recruiters of the PK (Figure 2A), affect trypanosomatid proliferation and genomic stability. In *Leishmania*, deletion of RAD50 can only be achieved in an MRE11 null mutant, suggesting an unanticipated, stoichiometric balance in activities provided by these two factors (Laffitte et al., 2016). Both factors operate during *Leishmania* HR, with MMEJ predominating in their absence, where chromosomal translocations are seen (Laffitte et al., 2016). In *T. brucei*, null mutants of either MRE11 or RAD50 are tolerated, with loss of the former leading to instability in the large, transcriptionally silent Variant Surface Glycoprotein (VSG) gene-rich subtelomeres (Robinson et al., 2002; Mehnert et al., 2021). Loss of either RAD50 or MRE11 results in increased levels of VSG activation after induction of a DSB within the specialized site for VSG transcription (termed the bloodstream expression site), whereas MRE11 mutants do not display such elevation in the rate of immune evasion without DSB induction (Robinson et al., 2002). Taken together, these data raise questions about how VSG-directed HR initiates during immune evasion (da Silva M. S. et al., 2018), and analysis of ATM could be key to understanding this reaction. Indeed, addressing ATM function may be informative in understanding signaling of gene family rearrangements (Weatherly et al., 2016) and gamma irradiation resistance (Regis-da-Silva et al., 2006) in *T. cruzi* and, perhaps, other trypanosomatids.

### DNA-PKcs: A *Leishmania*-Specific DDR PK?

Active DNA-PK is a holoenzyme complex consisting of DNA-PKcs and the Ku heterodimer (subunits Ku70 and Ku80 Gottlieb and Jackson, 1993). Together, this complex initiates DSB repair *via* cNHEJ. DNA-PK also shares partial functional redundancy with ATM; DNA-PK is capable of phosphorylating downstream ATM substrates, including H2AX, in cells lacking ATM (Stiff et al., 2004). DNA-PK can also orchestrate metabolic pathways like fatty acid synthesis (Chung, 2018). When a DSB forms, the Ku heterodimer recognizes the lesion and can recruit DNA-PKcs, which, in turn, is activated by autophosphorylation, forming the holoenzyme complex. DNA-PK phosphorylates and recruits downstream substrates to effect repair. First, mismatched ends of the DSB are resected by nucleases, followed by gap filling by DNA polymerases (mainly Pol μ and Pol ε), which act in a template-independent manner. Lastly, DNA ligase IV, in conjunction with the x-ray repair cross-complementing protein 4 (XRCC4) and the XRCC4-like factor (XLF), seals the break (reviewed by Chung, 2018; Mohiuddin and Kang, 2019; Menolfi and Zha, 2020). In recent years, a plethora of additional accessory NHEJ factors, such as the Paralog of XRCC4 and XLF (PAXX; previously known as C9orf142), have been discovered, though we are yet to comprehend the range of activities relating to NHEJ they perform (as reviewed by Ghosh and Raghavan, 2021). Insertions and deletions of the DNA template are frequent consequences of cNHEJ-directed repair. In some cases, such mutagenic repair is beneficial, such as when DNA-PK acts to generate antigen receptor diversity by coordinating Variable, Diverse, and Joining V(D)J recombination (Kienker et al., 2000). Thus, mutations in the DNA-PKcs gene in mice result in severe combined immune-deficiency (SCID) syndrome, manifesting as profound defects in T- and B-cell development. In humans, aberrant DNA-PK activity correlates with the development of a range of cancers (Mohiuddin and Kang, 2019).

Most kinetoplastids, including *T. brucei* and *T. cruzi*, appear to lack DNA-PKcs, whereas across *Leishmania* spp., a potential DNA-PKcs homolog has been identified (Figure 1). Putative DNA-PKcs homologs have also been found in the genomes of other *Leishmaniiae*, such as *Endotrypanun monterogeii* (a parasite of two-toed sloths) and *Crithidia* spp. (a monoxenous insect pathogen), but little is known about DNA repair in these organisms. The putative *Leishmania* DNA-PKcs shows most sequence conservation relative to other eukaryotic DNA-PKcs proteins within its C-terminal kinase domain. Additionally, a conserved NUC194 domain, whose function is unknown, has been identified in *Leishmania* DNA-PKcs, supporting this putative PK as a homolog of human DNA-PKcs (Lees-Miller et al., 2020). Functional analysis of this putative repair enzyme awaits and it is unknown if its loss alters the parasite’s response to genotoxic stress. The putative presence of DNA-PKcs in *Leishmania*, and other *Leishmaniiae*, unlike in related trypanosomatids, is especially intriguing because it is unlikely to direct cNHEJ since repair of CRISPR-Cas9-generated DSBs in *Leishmania* has never been shown to occur by this repair pathway, but instead only by MMEJ (Zhang and Matlashewski, 2015) or SSA (Zhang and Matlashewski, 2019).

Why *Leishmania* potentially possess DNA-PKcs poses another intriguing question since the Ku complex is present in *T. brucei* and *T. cruzi*, which have no ortholog of the putative DNA-PKcs gene. Addressing this complex pattern of presence or absence of components of the DNA-PK holoenzyme is further complicated by lack of clarity regarding what role Ku performs in the absence of cNHEJ, with the best evidence being a role in *T. brucei* telomere maintenance (Conway et al., 2002; Janzen et al., 2004), suggesting that this part of DNA-PK operates outside DSB repair in these parasites. The nature of this critical role remains unclear, given that the natural absence of both Ku proteins in *Blastocrithidia* spp. does not appear to have a noticeable impact on telomere length (Poláková et al., 2021). One possible explanation could be linked to the extensive genome plasticity observed in *Leishmania*, with aneuploidy (Sterkers et al., 2011) and copy number variations (CNVs) readily detected during growth (Ubeda et al., 2008; Leprohon et al., 2009; Rogers et al., 2011; Restrepo et al., 2019). Furthermore, the use of repair machinery for DNA replication (Damasceno et al., 2016, 2018, 2020) suggests that DNA repair processes are required for genome duplication. Thus, the presence of a putative complete DNA-PK in *Leishmania* but not in *T. brucei* or *T. cruzi* could play roles in genome maintenance and transmission that aid plasticity. For instance, the interaction between DNA-PKcs and Ku occurring at DSBs within unstable regions could activate a divergent DNA-PK pathway, perhaps amplifying repair by MMEJ or other more mutagenic pathways. Though *T. brucei* and *T. cruzi* also exhibit genomic instability, unstable regions in *T. brucei* appear limited to multicopy VSG gene families with functions in host immune evasion (Glover et al., 2013; Horn, 2014; Black et al., 2020). More widespread aneuploidy and CNVs have been reported in *T. cruzi* (Minning et al., 2011; Reis-Cunha et al., 2015; Callejas-Hernández et al., 2018), though the underlying mechanics are largely uncharacterized. Thus, *Leishmania* DNA-PKcs may perform *genus*-specific functions pertaining to plasticity, though further work is needed to demonstrate the presence and activity of the DNA-PK holoenzyme.

### The ATR Kinase

In most eukaryotes ATR is essential for cellular proliferation. For instance, during embryogenesis in mammals, loss of ATR results in mitotic catastrophe in the developing blastocyst (Brown and Baltimore, 2000). In adult mice, ATR depletion causes a premature aging-like syndrome that has been attributed to stem cell loss (Ruzankina et al., 2007) and appears akin to Seckel syndrome, a complex form of microcephalic primordial dwarfism that occurs in humans with ATR gene mutations (O’Driscoll et al., 2003). Interestingly, loss of ATR does not predispose such individuals to cancer, like loss of ATM (Chanan-Khan et al., 2003; Qvist et al., 2011). ATR is activated in response to ssDNA accumulation at stalled DNA replication forks, at resected DSBs, or following deoxyribonucleotide triphosphate (dNTP) depletion. Transcription-derived RNA-DNA hybrids (R-loops) and shortened telomeres are also prominent activators of ATR (reviewed by Saldivar et al., 2017). Briefly, ssDNA, coated with the heterotrimeric replication protein A (RPA) complex, acts as a recruitment platform for the obligatory interaction partner of ATR, ATR Interacting Protein (ATRIP; Figure 2A). ATRIP recruits and activates ATR, resulting in a hetero-tetrameric complex composed of two molecules each of ATR and ATRIP. Additionally, ATR activation requires the activities of the Rad9-Rad1-Hus1 (9-1-1) complex, topoisomerase II binding protein 1 (TOPBP1), and, in vertebrates, the Ewing tumor-associated antigen 1 (ETAA1; the latter two regulate the activity of ATR). Once activated, ATR phosphorylates the effector kinase checkpoint kinase 1 (CHK1), which initiates checkpoint activation and cell cycle arrest, suppressing global origin firing, promoting dormant origin firing, and initiating DNA repair pathways. Outside these DDR functions, ATR acts on centromeric R-loops to promote chromosome segregation during mitosis (Kabeche et al., 2018), on genome-wide R-loops to prevent instability (Matos et al., 2020), aids the replication of repetitive and fragile genomic regions (Casper et al., 2002), responds to mechanical stresses including nuclear and nucleolar deformation (Kidiyoor et al., 2016), and acts in telomere maintenance (McNees et al., 2010).

Like ATM, trypanosomatid ATR shares most sequence homology within the C-terminal kinase-containing region (Figure 1), and ∼70% of the enzymes are composed of an α-solenoid-like domain, which is typical of the PIKK family. Across all three trypanosomatids, FAT and FATC domains are present, in addition to an UME domain (NUC010; Pfam), which is characteristic of FAT and FATC domain-harboring proteins (the function of the UME domain is unknown). Intriguingly, trypanosomatid ATR appears to lack a PRD domain typical of PIKK kinases. This absence may be mechanistically important since the PRD domain is required for ATR activation by TOPBP1 (Mordes et al., 2008). Though a putative trypanosomatid homolog of TOPBP1 has been identified, its function remains uninvestigated (Genois et al., 2014) and no interactions between parasitic ATR and this putative TOPBP1 homolog have been reported. In mammalian-infective *T. brucei*, depletion of ATR produces an accumulation of cells in the S-phase accompanied by growth arrest, indicating that PK is essential even *in vitro* (Jones et al., 2014; Black et al., 2020). Depletion of ATR also resulted in widespread accumulation of genotoxic stress markers, including increased levels of yH2A and formation of RAD51 and RPA foci, and increased sensitivity to a range of DNA mutagens (Black et al., 2020) implicating ATR in the trypanosomatid DDR. Nonetheless, what aspect of ATR function results in *T. brucei* death after the loss of the PK is unknown. In this regard, recent work in insect stage *T. brucei* revealed that depletion of ATR only moderately affects parasite proliferation and cell cycle progression, despite playing an important role during HR and damage signaling in this life cycle stage in response to ionizing radiation (IR) (Marin et al., 2020). This dichotomy likely reflects alternative demands on repair and replication in distinct life cycle stages.

A parasite-specific and life cycle stage-specific role of ATR has been uncovered in mammalian-infective *T. brucei*. To evade immune clearance, stochastic switching of the VSG surface antigen occurs. On any cell, at any given time, a single VSG variant is expressed out of the predicted 2,000 VSGs available in the genome, the majority of which comprise a subtelomeric library (Müller et al., 2018). VSGs are transcribed by polymerase I (Navarro and Gull, 2001; Hertz-Fowler et al., 2008) from a specialized subtelomeric expression site known as the Bloodstream Expression Site (BES), of which ∼15 have been reported in the laboratory-adapted Lister 427 strain (Müller et al., 2018). Upon ATR depletion, downregulation of the actively transcribed BES occurs, correlating with increased transcription from previously silent BESs, indicating that loss of ATR undermines BES transcriptional control. Furthermore, transcripts from VSGs located in the subtelomeric library became upregulated, suggesting increased levels of recombination events moving these VSGs into BESs. Perhaps explaining both these effects on VSG expression, increased damage was detected across the majority of BESs and in close proximity to the VSG-associated 70-bp repeats, implying that ATR may play a role in the resolution of lesions that accumulate within the BES. One possible form of BES lesion is an R-loop since these structures have been shown to accumulate in BESs after the loss of RNase H enzymes, leading to the same changes in VSG expression (Briggs et al., 2018a, 2019). Nonetheless, how ATR (and potentially R-loops) acts in VSG transcriptional control and VSG recombination remains unclear (Black et al., 2020).

In *Leishmania*, ATR function has been investigated in promastigote cells using the small-molecule inhibitor VE-821 (da Silva R. B. et al., 2018), a selective inhibitor of the ATR kinase in humans (Charrier et al., 2011; Reaper et al., 2011). VE-821 treatment was associated with a modest decrease in proliferation, though no cell cycle alteration was reported, as seen following ATR depletion in *T. brucei*. However, as for ATM, VE-821-treated cells were significantly more sensitive to H_2_O_2_, suggesting that ATR may act during the response to oxidative stress, similar to ATM, though work is needed to validate ATR as the target of VE-821 and to assess whether inhibiting ATR also sensitizes cells to other genotoxins. Unlike in *T. brucei*, and perhaps consistent with the VE-821 inhibition data, ATR has been reported to be dispensable for *L. mexicana* survival *in vitro*, though effects of ATR loss were not investigated further (Baker et al., 2021).

A major deficit in our understanding of the trypanosomatid ATR pathway is the initial activation of ATR itself. Other factors operating within the ATR pathway include the 9-1-1 complex, which has been functionally characterized in *L. major*, revealing connections between DNA signaling pathways with genome plasticity (Damasceno et al., 2018). How ATR interacts with 9-1-1 in these organisms is unknown. Given that Rad9 likely operates as part of an alternative complex to 9-1-1, and Hus1 is capable of persisting in a monomeric form (Damasceno et al., 2016), such interactions may be divergent and parasite-specific. Does ATR interact with both complexes? Does ATR modulate their behavior or do they modulate the behavior of ATR?

The genomes of all trypanosomatids also appear to lack any putative homologs of the obligatory ATR interaction partner ATRIP (or ETAA1), which is required for kinase activation in other eukaryotes. We also lack information on the roles of the putative TopBP1 homolog (Genois et al., 2014), which is a critical ATR activation factor (Kumagai et al., 2006) and a recruiter of the 9-1-1 complex, *via* Rad9 (Yan and Michael, 2009). TopBP1 in other eukaryotes interacts with ATR *via* a small domain, the PIKK regulatory domain (PRD), upstream of the FATC domain; PRD deletion prevents ATR activation by TopBP1. The PRD domain of trypanosomatid ATR is less well defined and, when combined with poor conservation of the ATR activation domain (AAD) in the trypanosomatid TopBP1 homologs, this raises questions as to whether TopBP1 plays a role in ATR kinase activation. Functions of the RPA complex also raise questions about the activation of trypanosomatid ATR. Trypanosomatid RPA1 can bind to the ends of telomeres and may regulate telomere homeostasis (Pavani et al., 2016, 2018; Fernandes et al., 2020). In other eukaryotes, ATR functions to stabilize telomeres (McNees et al., 2010): ATR loss associated with R-loop and G4 structure accumulation destabilizes these structures, resulting in telomere dysfunction (Rhodes and Lipps, 2015; Graf et al., 2017). Given RPA activates ATR, it is possible that the kinase acts directly at the telomeres of trypanosomatids. In support of this, loss of ATR is linked to damage accumulation within subtelomeric regions in *T. brucei* correlating with regions of R-loop formation (Briggs et al., 2018a, b; Black et al., 2020). The functions of ATR in *T. cruzi* are unknown.

### DDR Effector Kinases: What Goes on Downstream?

ATR, ATM, and DNA-PKcs, and their direct downstream substrates (discussed above), are key DDR players, coordinating much of the initial response to a DNA lesion. However, a plethora of other PKs also act in a wider response to restore cellular homeostasis after damage. In humans, up to 160 PKs (out of ∼550 PKs encoded in the genome; Eid et al., 2017; Kanev et al., 2019) have been linked to neoplastic cellular transformation or disease development due to mutations causing a loss or gain of function (Cell Signaling Technology, 2020). Assessing wider damage response functionality has been made possible through the use of systematic high-throughput screening using siRNAs, small-molecule inhibitors, or CRISPR/Cas9 technology to identify novel DDR factors and map damage response pathways across several eukaryotic organisms. Indeed, a recent genome-wide screen performed in the presence or absence of a panel of genotoxins has revealed ∼890 genes that may function during DNA repair in human cells, including ∼40 PKs (based on GO term analysis of hits on protein serine/threonine kinase activity; Olivieri et al., 2020).

*T. brucei*, *T. cruzi*, and *Leishmania* encode for 190 (Jones et al., 2014), 190 (Parsons et al., 2005), and 206 (Baker et al., 2021) PKs, respectively, with several aPKs identified and members of all ePK groups represented, except for tyrosine-like and tyrosine kinases (Figure 2B). Over the last decade, with the implementation of genome-wide and kinome-focused screens in *T. brucei*, the roles of PKs have been investigated during drug resistance (Alsford et al., 2012), cell cycle control (Jones et al., 2014), and *in vivo* survival (Fernandez-Cortes et al., 2017). However, only one screen to date has been performed to examine the parasite’s response to DNA damage. Both genome-wide and kinome-focused RNAi screening identified a cohort of 30 PKs (∼15% of the kinome), whose downregulation was associated with increased sensitivity to MMS. Among these 30 PKs, and in addition to ATR, ATM, and the related kinase TOR4, eight novel putative DDR PKs were validated. Within this cohort was AUK2 (Stortz et al., 2017), a member of the aurora kinase family, and homologous to AURKA in human cells (the function of aurora kinases is reviewed here; Tang et al., 2017; Willems et al., 2018). Deletion of AUK2 in *T. brucei* resulted in increased DNA damage sensitivity, cell cycle defects, spindle formation defects, yH2A phosphorylation, and RAD51 foci formation, indicating the accumulation of DNA lesions and highlighting AUK2 as a DDR kinase. AUK2 is also required for the survival of *in vivo* murine infections (Fernandez-Cortes et al., 2017). Dysregulation of aurora kinase family members is associated with the formation of cancer, and the PKs play prominent roles during mitosis (Tang et al., 2017). The function of AUK2 is unknown in *Leishmania*, but null mutants could not be recovered in promastigote cells, suggesting that it is essential (Baker et al., 2021). In *T. cruzi*, only AUK1 (homologous to AURKB) function has been assessed, with evidence suggesting it acts canonically during mitosis and nuclear division, alongside being required during kinetoplast duplication (Fassolari and Alonso, 2018). Though the role of AUK2 was not directly investigated in *T. cruzi*, the authors reported two independent forms of the protein, suggesting that this kinase may functionally diverge from AURKA, and indeed may display variation in AUK2 functions in *T. brucei*.

From the genome-wide screen (Stortz et al., 2017), the tousled-like kinases 1 and 2 (TLK1/2) were identified as causing increased MMS sensitivity following their simultaneous depletion by RNAi. RNAi depletion resulted in a loss of proliferation, an S-phase stall, increasing numbers of cells lacking nuclear DNA (indicating nuclear segregation defects), and increased phosphorylation of yH2A (Stortz et al., 2017). Indeed, earlier RNAi implicated TLK1 as the perpetrator of these defects, with TLK1 localizing to the nucleus of the parasites (Li et al., 2007). In metazoans, TLKs can act during genome maintenance, in keeping with the role of TLK1 reported for *T. brucei*. TLK is an essential gene in *Leishmania*, likely controlling aspects of the cell cycle, though DDR-related roles have not been described (Baker et al., 2021). Across both screens, further investigation of candidate DDR PKs, in addition to AUK2, revealed a further four whose loss causes increased sensitivity to MMS, but no proliferative defects were detected upon RNAi *in vitro*, suggesting that these kinases are required for parasite survival specifically following genotoxic stress exposure. These four PKs belong to diverse PK families, including calmodulin-dependent protein kinases (CAMK), which act to regulate intracellular calcium stores including during apoptosis, and the CMGC family, which include regulators of cell cycle progression. In *Leishmania*, these four PKs are non-essential, as CRISPR/Cas9-mediated null mutants are viable *in vitro* (Baker et al., 2021). Further work will be needed to investigate the role of these enzymes in the DDR, including asking how they map onto the pathways elicited by ATM, ATR, and, perhaps, DNA-PKcs. In *L. mexicana*, the recently developed CRISPR/Cas9 bar-seq library could be used to perform the type of DDR screen performed in *T. brucei*. In addition, single-cell transcriptomics may be a key strategy to examine the timing of PK expression during parasite growth, as well as to map the interacting PK signaling activities. In *T. cruzi*, no genome-wide libraries are currently available but the recent introduction of the CRISPR/Cas9 system in this parasite (Lander et al., 2015; Peng et al., 2015) could mean such screens are on the horizon.

## Conclusion and Future Perspectives

Genome integrity must be preserved to prevent loss of information across generations. PKs are key facilitators of this process and their integral roles across a multitude of DDR pathways make them opportune candidates for drug development pipelines. For trypanosomatids, where many aspects of core biology are diverged, focused and broad approaches to study DDR PK functions have revealed novelty, such as the participation of ATR in host immune evasion in *T. brucei*, and the proposed role for ATM in *Leishmania* development in the insect vector. In contrast, we lack information about the DDR PK function in *T. cruzi*. Continued forays into trypanosomatid PK function provide the prospect of new drug targets, by re-purposing available small-molecule inhibitors, and could offer tantalizing glimpses into the evolution of core biological processes in these peculiar eukaryotes.

## Author Contributions

All authors listed intellectually contributed to the review, preparation, and submission of this article. All figures in this review were generated using BioRender.com.

## Conflict of Interest

The authors declare that the research was conducted in the absence of any commercial or financial relationships that could be construed as a potential conflict of interest. The reviewer VY declared a past co-authorship with one of the author RM to the handling editor.

## Publisher’s Note

All claims expressed in this article are solely those of the authors and do not necessarily represent those of their affiliated organizations, or those of the publisher, the editors and the reviewers. Any product that may be evaluated in this article, or claim that may be made by its manufacturer, is not guaranteed or endorsed by the publisher.
